# CX3CR1–TLR4 Axis as a Shared Neuroimmune Target in COVID-19 and Epilepsy: Integrative Transcriptomics and Gabapentin Repositioning

**DOI:** 10.3390/biomedicines13092133

**Published:** 2025-08-31

**Authors:** Nannan Pan, Penghui Cao, Ben Chen, Li Chen, Xuezhen Liao, Yuping Ning

**Affiliations:** 1 Department of Neurology, Affiliated Brain Hospital of Guangzhou Medical University, Guangzhou 510370, China; 2024360094@gzhmu.edu.com (N.P.);; 2 Department of Psychiatry, The Fifth Affiliated Hospital of Sun Yat-sen University, Zhuhai 519000, China; 3 Geriatric Neuroscience Center, Affiliated Brain Hospital of Guangzhou Medical University, Guangzhou 510370, China; 4 Key Laboratory of Neurogenetics and Channelopathies of Guangdong Province and the Ministry of Education of China, Guangzhou Medical University, Guangzhou 511436, China

**Keywords:** epilepsy, COVID-19, CX3CR1, TLR4, neuroinflammation, gabapentin

## Abstract

**Introduction:** Neuroinflammation is a common pathological hallmark of Coronavirus Disease 2019 (COVID-19) and epilepsy; however, their shared immunogenomic mechanisms remain poorly defined. This study explores shared immune-inflammatory transcriptomic signatures and identifies potential repositioning therapeutics. **Methods:** We integrated single-cell RNA-seq data from peripheral blood mononuclear cells (PBMCs) of COVID-19 patients and healthy donors (GSE149689), and bulk RNA-seq data from hippocampal tissue of patients with Temporal Lobe Epilepsy with Hippocampal Sclerosis (TLE-HS) and healthy controls (GSE256068). Common Differentially Expressed Genes (DEGs) were identified and subjected to GO/KEGG enrichment, a PPI network, hub gene detection (cytoHubba), and transcriptional regulation analysis (ENCODE-based TF/miRNA networks). Drug repositioning was performed using the LINCS L1000 database. **Results:** We identified 25 DEGs shared across datasets, including 22 upregulated genes enriched in cytokine–cytokine receptor interaction, NF-κB, and Toll-like receptor pathways. PPI analysis revealed a CX3CR1–TLR4-centered immune module. Gabapentin emerged as a promising repositioning candidate with potential to downregulate CX3CR1, TLR4, and selectin P ligand (SELPLG). Receiver Operating Characteristic (ROC) analysis confirmed the diagnostic value of these targets (AUC > 0.90 in epilepsy). A mechanistic model was proposed to illustrate Gabapentin’s dual action on microglial polarization and cytokine suppression. **Conclusions:** Our results reveal a shared CX3CR1–TLR4–NF-κB inflammatory axis in COVID-19 and epilepsy, supporting Gabapentin as a potential dual-action immunomodulator. These findings reveal a previously underappreciated immunomodulatory role for Gabapentin, providing mechanistic rationale for its repositioning in neuroinflammatory conditions beyond seizure control.

## 1. Introduction

Epilepsy is a chronic neurological disorder characterized by recurrent seizures, affecting approximately 70 million people globally and posing substantial clinical and public health burdens [1,2]. Among various subtypes, TLE-HS is particularly challenging due to its frequent drug-resistance and significant cognitive impairment [3,4]. In recent years, immune and inflammatory mechanisms—such as microglial activation, cytokine dysregulation, and disruption of blood–brain barrier (BBB) integrity—have emerged as crucial contributors to epileptogenesis, especially in TLE-HS [5,6,7]. Moreover, peripheral immune dysfunction has increasingly been implicated in seizure initiation and progression, suggesting a broader immunopathological framework beyond the central nervous system. Indeed, inflammatory mediators released by both brain-resident cells and circulating immune cells have been shown to contribute to epileptogenesis [8].

COVID-19 pandemic, caused by severe acute respiratory syndrome coronavirus 2 (SARS-CoV-2), has revealed profound interactions between viral infection, systemic inflammation, and neurological dysfunction. Patients with severe COVID-19 often present with neurological symptoms—including seizures, encephalopathy, and cognitive deficits—even when direct viral neuroinvasion is absent [9,10]. These neurological manifestations are commonly attributed to hyperinflammatory conditions, including cytokine storms and endothelial dysfunction, potentially exacerbating neuroimmune dysregulation [11,12].

Notably, clinical reports have documented new-onset seizures or exacerbation of pre-existing epilepsy in patients hospitalized with severe SARS-CoV-2 infection, often accompanied by worse neurological prognosis [13,14]. Such manifestations underscore systemic hyperinflammation’s role in promoting neuroimmune dysregulation and seizure susceptibility [9,15].

Despite these converging lines of clinical and mechanistic evidence, it remains unclear whether COVID-19 and epilepsy share common immune-related transcriptional programs. Previous studies have primarily examined each disease in isolation, lacking integrative insight into their shared molecular architecture. Elucidating overlapping immunopathological pathways could not only clarify the neurological complications of COVID-19 but also reveal novel therapeutic avenues for epilepsy [16].

**Figure 1:**
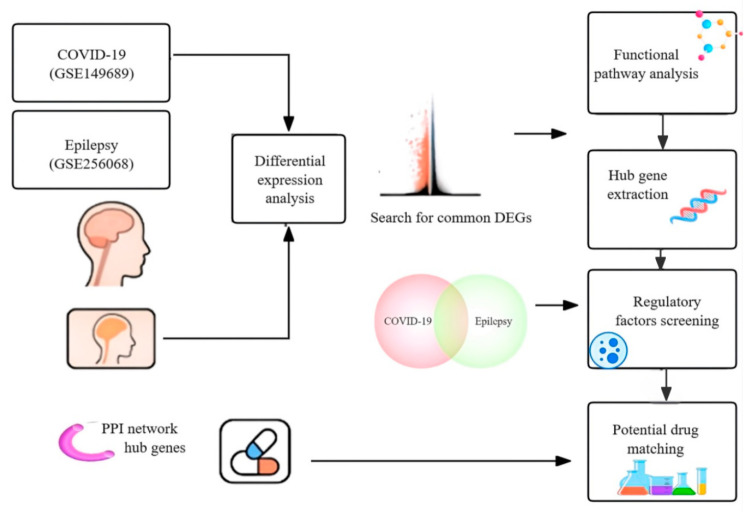
Schematic overview of the integrative transcriptomic analysis workflow. Shared DEGs between COVID-19 and epilepsy were identified from RNA-seq datasets and analyzed by pathway enrichment, hub gene extraction, regulatory network construction, and drug repositioning.

To address this knowledge gap, we conducted a comprehensive transcriptomic integration of two public datasets: single-cell RNA sequencing (scRNA-seq) data from peripheral blood mononuclear cells (PBMCs) of COVID-19 patients (GSE149689) and bulk RNA-seq data from hippocampal samples of TLE-HS patients (GSE256068). We identified shared DEGs, performed functional enrichment analyses (GO and KEGG), constructed protein–protein interaction (PPI) and transcription factor–miRNA (TF–miRNA) networks, and applied perturbation-based drug repositioning via the LINCS L1000 platform. Among several repositioning candidates, Gabapentin emerged unexpectedly as a dual-function agent with the potential to modulate both neuronal excitability and immune polarization. This study offers novel insight into convergent neuroimmune mechanisms and proposes a rational strategy for therapeutic repurposing in inflammatory epilepsy and COVID-19-associated neurological disorders. A schematic overview of the analytic pipeline is illustrated in Figure 1.

## 2. Materials and Methods

### 2.1. Patient Inclusion Criteria and Sample Characteristics

For the COVID-19 dataset (GSE149689), we specifically analyzed scRNA-seq data from peripheral blood mononuclear cells (PBMCs) of patients with PCR-confirmed COVID-19 and healthy donors. Samples from influenza patients were excluded. Only COVID-19 (mild and severe cases) and healthy control groups were included in the present study. Clinical and demographic details are available in the original publication.

For the epilepsy dataset (GSE256068), we included hippocampal tissue samples from patients with TLE-HS and age- and region-matched controls. All cases were independently reviewed by two neuropathologists, and the diagnosis of hippocampal sclerosis was made according to the International League Against Epilepsy criteria. Patients with previous resective epilepsy surgery or invasive electrode implantation were excluded. Control hippocampal tissues were obtained at autopsy from individuals without neurological disease, with causes of death including arrhythmia, myocardial infarction, and acute cardiorespiratory failure. All autopsies were conducted within 12 h of death, and tissue handling followed local ethical guidelines and the Declaration of Helsinki.

### 2.2. Transcriptomic Datasets and DEG Analysis

To identify shared immune-inflammatory signatures between COVID-19 and epilepsy, we analyzed two Gene Expression Omnibus (GEO) datasets. GSE149689 comprises scRNA-seq data generated from PBMCs of COVID-19 patients and uninfected controls. GSE256068 provides bulk RNA-seq data from hippocampal tissue of patients with TLE-HS and healthy controls.

For GSE149689, raw 10X Genomics files were processed in Seurat (v4.2.2; Boston, MA, USA), a widely used R toolkit for single-cell transcriptomic analysis. Cells with fewer than 200 detected genes were removed. To reduce cell-type variability, we aggregated counts by donor (pseudo-bulk), followed by log_2_ transformation with a pseudocount of 1. Group labels (“COVID-19” vs. “Normal”) were assigned based on metadata.

For the GSE256068 dataset, we utilized the authors’ published differential expression results without further normalization or batch correction due to unavailable batch metadata.

DEGs were identified using FindMarkers() (Wilcoxon test) in Seurat for GSE149689 and from DESeq2 (Bioconductor, USA) results for GSE256068. Genes with an adjusted *p*-value < 0.05 and a |log_2_FC| > 1 were considered significant. Ensembl IDs were mapped to gene symbols via the org.Hs.eg.db package (Bioconductor, USA). After filtering, we identified 25 shared DEGs (22 upregulated, 3 downregulated). A Venn diagram was generated to illustrate the overlap. The complete lists of shared upregulated and downregulated DEGs with statistical details are provided in Supplementary Files S1 and S2.

### 2.3. Functional Enrichment Analysis (GO and KEGG)

We performed Gene Ontology (GO) and KEGG pathway enrichment for the shared DEGs using clusterProfiler (v4.6.2; Bioconductor, USA). GO terms were grouped into Biological Process (BP), Cellular Component (CC), and Molecular Function (MF). KEGG annotations used the *human* “hsa” tag.

Gene symbols were converted to Entrez IDs via org.Hs.eg.db (Bioconductor, USA) and biomaRt (Bioconductor, USA). Terms with an adjusted *p* < 0.05 were considered significant. Dot plots were used to visualize enriched pathways for upregulated and downregulated genes separately. The full enrichment results are provided in Supplementary Files S3 and S4.

### 2.4. Protein–Protein Interaction (PPI) Network and Hub Gene Identification

We built protein–protein interaction networks for the shared DEGs using the STRING database (v11.5; EMBL, Heidelberg, Germany), with a medium confidence threshold (0.4). Unconnected nodes and self-loops were removed.

Networks were imported into Cytoscape (v3.9.1; San Diego, CA, USA). Hub genes were identified using the Maximal Clique Centrality (MCC) algorithm in cytoHubba (San Diego, CA, USA). To find functional clusters, MCODE (San Diego, CA, USA) was used, with degree cutoff = 2, node score cutoff = 0.2, K-core = 2, and max depth = 100. These clusters helped guide further analysis. The complete PPI edge tables, hub-gene rankings, and MCODE clusters are provided in Supplementary Files S5–S7.

### 2.5. Pathway-Level Immune Convergence

For each dataset, we performed gene set enrichment analysis (GSEA; Cambridge, MA, USA) to assess shared immune pathways. All genes were ranked by log_2_ fold change (COVID-19) or Wald statistic (epilepsy). A custom TERM2GENE table was constructed using immune-related pathways curated from Reactome (Toronto, ON, Canada), KEGG (Kyoto, Japan), and published sources [3]. GSEA was run with 1000 permutations, a minimum gene set size of 3, and a false discovery rate (FDR) < 0.05. Normalized enrichment scores (NESs) were used to quantify pathway activation.

### 2.6. Regulatory Network Construction: TF and TF-miRNA

To explore regulatory mechanisms behind the shared immune profile, we built transcription factor (TF) and TF–miRNA co-regulatory networks. TF–gene links were identified using ENCODE ChIP-seq data (Santa Cruz, CA, USA) in NetworkAnalyst (Toronto, ON, Canada). TF–miRNA–gene networks used a combination of ENCODE TF binding and miRTarBase (Taipei, Taiwan) miRNA–target interactions.

Up- and downregulated genes were analyzed separately to highlight different regulatory trends linked to immune activation or resolution. To focus on the core structure, nodes with Degree < 2 were excluded from visualization and node analysis. The underlying TF–gene and TF–miRNA interaction datasets are provided in Supplementary Files S8 and S9.

### 2.7. Drug Repositioning Analysis and Target Validation

We used Enrichr (New York, NY, USA)to identify drugs that could reverse the disease-related gene expression patterns. DEGs were matched against Drug Signatures Database (DSigDB; Aurora, CO, USA), DrugMatrix (NC, USA), and the LINCS L1000 Chemical Perturbation (CP) dataset (Cambridge, MA, USA). LINCS compares disease gene signatures with compound-induced profiles.

Drugs with an adjusted *p* < 0.05 and strong reversal scores were shortlisted. Candidates were filtered based on literature showing immune or neural effects. We used ROC analysis to evaluate the predictive value of drug-targeted genes (AUC > 0.70 considered informative). Gene expression trends were also checked across both datasets.

### 2.8. Literature-Based Validation of Gabapentin Targets

Gabapentin, one of the top repositioning hits, was further assessed for effects on both inflammation and seizure activity. We reviewed published studies that used Gabapentin and measured outcomes related to immune pathways (e.g., CX3CR1, TLR4, NF-κB) or neuronal excitability.

Findings included a meta-analysis confirming Gabapentin’s effectiveness in focal epilepsy and several preclinical studies showing its role in reducing microglial activation through high-mobility group box 1 (HMGB1)–Toll-like receptor 4 (TLR4) and CX3CL1–CX3CR1 signaling. These results support its dual role in modulating neuroinflammation and hyperexcitability. Key studies are listed below. No further computational or lab-based validation was performed.

## 3. Results

### 3.1. Identification of Shared Differentially Expressed Genes Between COVID-19 and Epilepsy

We identified 2571 upregulated and 733 downregulated genes in peripheral blood mononuclear cells (PBMCs) from COVID-19 patients (GSE149689). In the epilepsy dataset (GSE256068), 173 genes were upregulated and 363 were downregulated in hippocampal samples.

**Figure 2:**
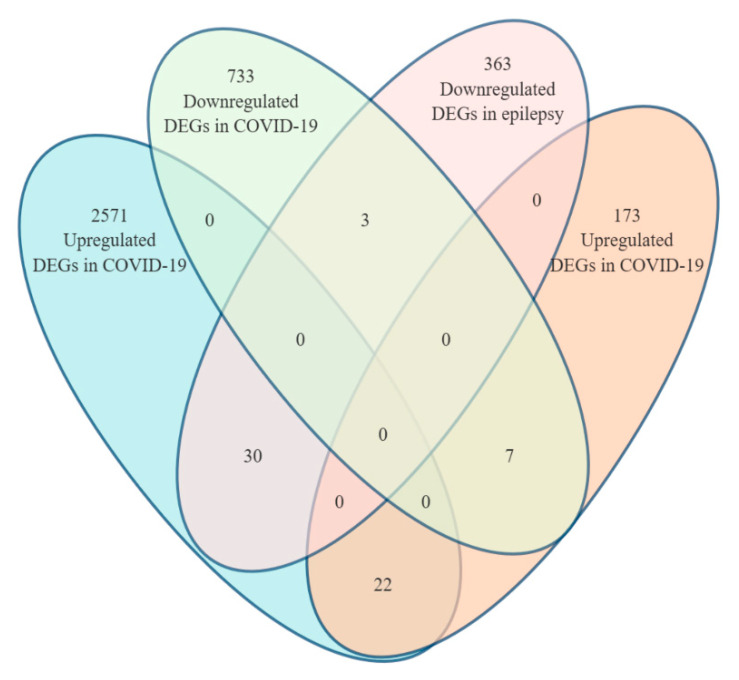
Venn diagram depicting the overlap of significantly upregulated and downregulated DEGs between COVID-19 (GSE149689) and epilepsy (GSE256068).

A comparison of both datasets revealed 25 shared DEGs, including 22 that were consistently upregulated and 3 that were consistently downregulated (Figure 2).

Shared upregulated genes included critical neuroinflammation mediators (e.g., *TNF*, *CCL3*, *CXCL10*, *CX3CR1*, *CD69*), involved in cytokine signaling, immune cell recruitment, and glial activation. In contrast, the downregulated genes—*PECAM1*, *FCGR3A*, and *CR1*—are linked to vascular adhesion and immune effector clearance, suggesting impaired resolution of inflammation.

These overlapping DEGs were used in subsequent GO and KEGG enrichment analyses to explore common immune-related mechanisms in both conditions.

### 3.2. Functional Enrichment Analysis of Common DEGs Between COVID-19 and Epilepsy

**Figure 3:**
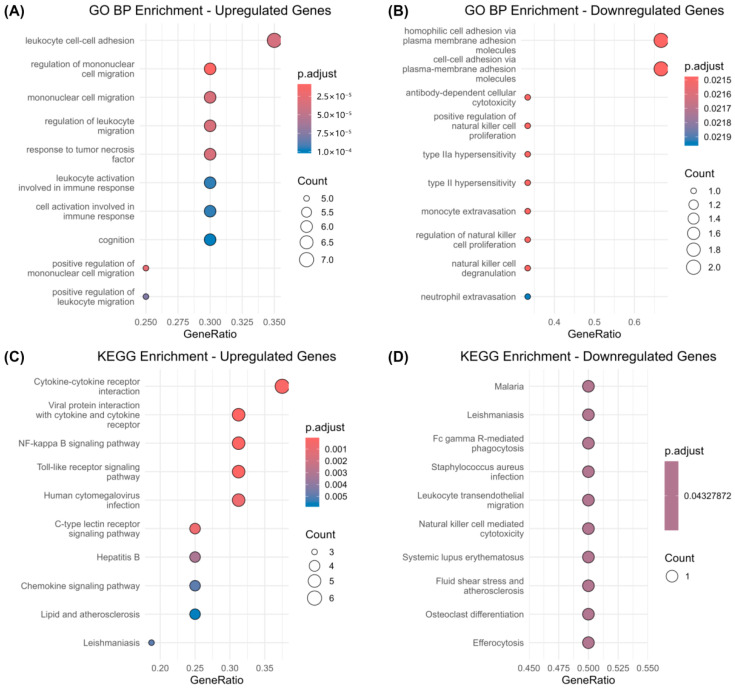
Functional enrichment analysis of shared DEGs. (**A**,**B**) GO biological processes enriched among commonly upregulated (**A**) and downregulated (**B**) genes. (**C**,**D**) KEGG pathways enriched among upregulated (**C**) and downregulated (**D**) DEGs.

GO enrichment of the 22 upregulated genes showed significant involvement in processes such as leukocyte adhesion, mononuclear cell migration, and TNF-related inflammatory responses (Figure 3A). These findings suggest increased immune cell recruitment and vascular inflammation.

In contrast, downregulated genes were mainly associated with cytotoxic immune functions, including natural killer (NK) cell activity and antibody-dependent cellular cytotoxicity (Figure 3B), indicating impaired immune clearance mechanisms.

KEGG analysis corroborated these findings. Upregulated genes were enriched in pathways such as cytokine–cytokine receptor interaction, NF-κB signaling, and Toll-like receptor signaling (Figure 3C). Downregulated genes were involved in pathways related to Fc gamma receptor-mediated phagocytosis, NK cell cytotoxicity, and transendothelial migration (Figure 3D).

Taken together, the data point to a two-sided immune imbalance: overactivation of inflammatory pathways alongside weakened resolution mechanisms. This dual pattern may underlie sustained neuroinflammation in both conditions.

### 3.3. Protein–Protein Interaction (PPI) Network Analysis of Common DEGs

**Figure 4:**
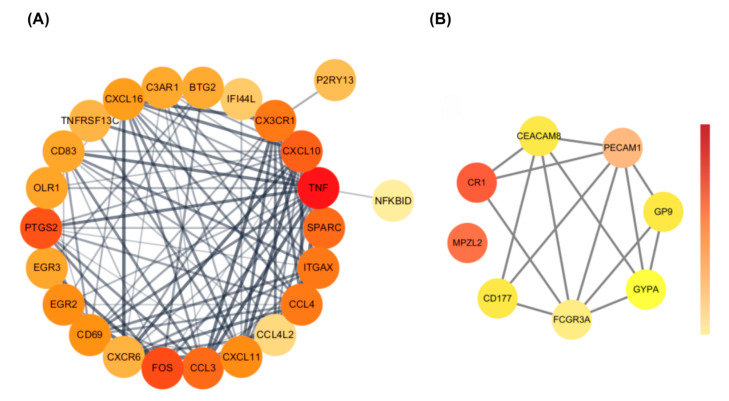
PPI networks of shared DEGs. (**A**) Upregulated genes formed a densely connected inflammatory module. (**B**) Downregulated genes clustered around immune effector functions. Node color reflects closeness centrality.

PPI analysis revealed distinct network patterns between upregulated and downregulated genes (Figure 4). The upregulated genes formed a densely connected proinflammatory module, with *TNF*, *CXCL10*, *CCL3*, and *CX3CR1* at the center. These genes are known to drive cytokine signaling, microglial activation, and immune cell recruitment.

In contrast, the network for downregulated genes was more loosely connected. It included *PECAM1*, *FCGR3A*, and *CEACAM8*, which are involved in maintaining vascular structure and supporting immune clearance. The weaker connectivity here suggests reduced activity in resolution-phase immune responses.

Overall, these opposing network structures reflect an immune imbalance—heightened inflammatory signaling alongside diminished regulatory function—which may contribute to ongoing neuroinflammation in both conditions.

### 3.4. Identification of Hub Genes

**Figure 5:**
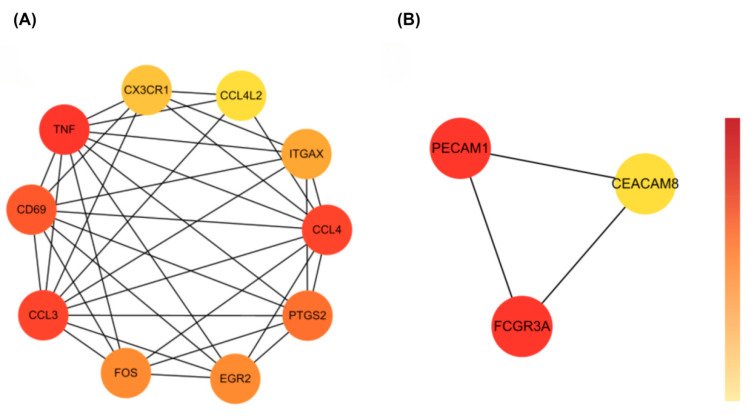
Hub gene networks derived from shared DEGs. (**A**) Upregulated hub genes identified by maximal clique centrality (MCC). (**B**) Downregulated hub genes identified by MCC analysis. Node color indicates MCC score.

To pinpoint key regulators within the shared immune signature, we applied Maximal Clique Centrality (MCC) analysis using the cytoHubba plugin in Cytoscape. For the upregulated genes, ten hub genes were identified: *TNF*, *CX3CR1*, *CCL4L2*, *integrin subunit alpha X* (*ITGAX*), *CCL4*, *CD69*, *CCL3*, *FOS*, *EGR2*, and *PTGS2* (Figure 5A). These genes formed a tightly connected proinflammatory module involved in cytokine signaling, immune cell recruitment, and glial activation.

Among them, *FOS* and *EGR2* are transcription factors likely involved in rapid immune responses, while TNF and *CX3CR1* are central players in neuroimmune amplification.

For the downregulated set, the hub genes *PECAM1*, *FCGR3A*, and *CEACAM8* were identified (Figure 5B). These genes are known for their roles in maintaining vascular stability and supporting immune effector clearance. Their reduced expression may point to impaired inflammation resolution and disrupted neurovascular regulation.

Together, these hub networks suggest a two-sided immune imbalance—amplified proinflammatory signaling on one hand and weakened immune resolution on the other—which may contribute to the chronic neuroinflammation observed in both COVID-19 and epilepsy.

### 3.5. Gene Set Enrichment Analysis Reveals Immune Convergence in Cytokine Signaling

**Figure 6:**
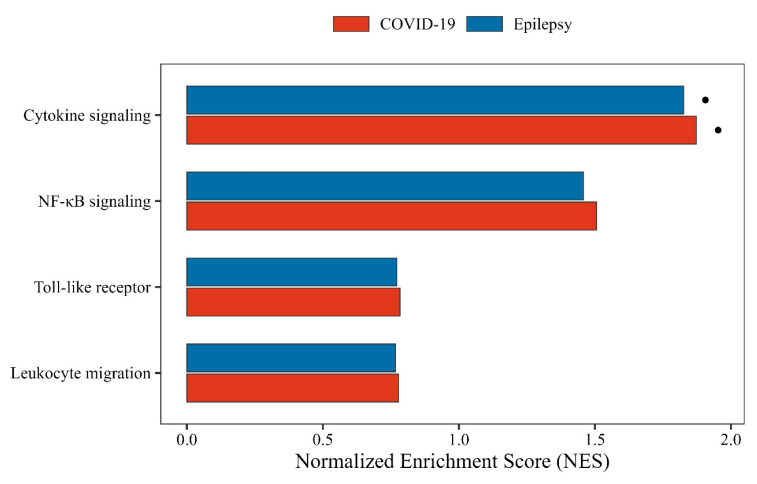
Gene set enrichment analysis (GSEA) of four shared immune-related pathways. Normalized enrichment scores (NESs) are shown for COVID-19 and epilepsy. Cytokine signaling was the only pathway significantly enriched in both conditions (*FDR* < 0.05), whereas NF-κB signaling, Toll-like receptor signaling, and leukocyte migration did not reach statistical significance (*FDR* > 0.05). Exact NES and FDR values are indicated. The small black dots at the ends of the bars represent *FDR* < 0.05, indicating statistical significance.

We performed gene set enrichment analysis (GSEA) to evaluate shared immune pathways between COVID-19 and epilepsy. Among the tested signatures, cytokine signaling was the only pathway significantly enriched in both datasets—COVID-19 (NES = 1.87, FDR = 0.01) and epilepsy (NES = 1.83, FDR = 0.01) (Figure 6).

By contrast, NF-κB signaling, Toll-like receptor signaling, and leukocyte migration exhibited only modest enrichment and did not reach statistical significance (FDR > 0.05).

These findings highlight cytokine signaling as the major area of immune convergence between the two conditions, consistent with our KEGG enrichment and hub-gene analyses, in which *TNF*, *CCL3*, and *CX3CR1* were prominently involved. The shared activation of this pathway supports a central role for cytokine-driven inflammation in both COVID-19-related brain dysfunction and epilepsy-associated immune dysregulation.

### 3.6. Transcriptional Regulatory Network Analysis

To explore upstream regulation of the shared immune signature, we built transcription factor (TF)–gene interaction networks using ENCODE ChIP-seq data through NetworkAnalyst. Separate networks were constructed for upregulated and downregulated hub genes.

**Figure 7:**
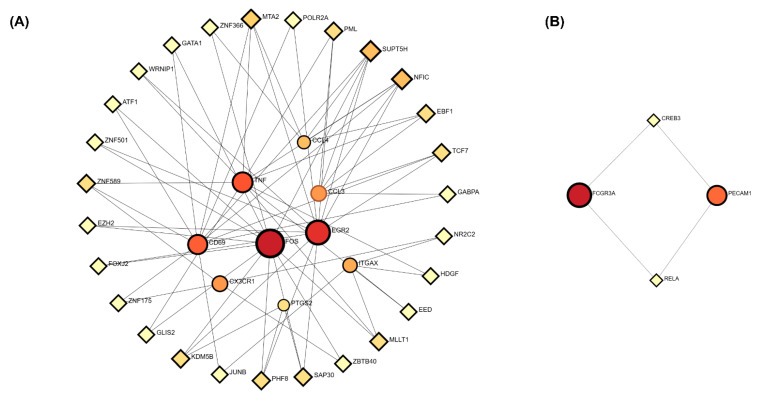
Transcriptional regulatory networks of shared hub genes in COVID-19 and epilepsy. (**A**) Network of transcription factors (TFs, diamonds) and upregulated hub genes (circles). Node size and color indicate regulatory degree, highlighting *FOS*, *EGR2*, and *ZNF589* as key transcriptional regulators. (**B**) Downregulated gene network with *PECAM1* and *FCGR3A* as central nodes, regulated by RELA and CREB3, suggesting potential suppression of immune effector functions.

In the upregulated network (Figure 7A), *FOS*, *EGR2*, and *ZNF589* stood out as key TFs targeting genes such as *TNF*, *CX3CR1*, and *CD69*. These factors are linked to cytokine signaling and glial activation, suggesting a transcriptional circuit that supports inflammatory responses.

The network for downregulated genes (Figure 7B) was more limited, with *RELA* and *CREB3* identified as regulators of *PECAM1* and *FCGR3A*. This pattern may indicate reduced activation of pathways related to vascular integrity and immune clearance.

Together, these opposing regulatory trends—activation of proinflammatory TFs and suppression of resolution-linked TFs—may contribute to sustained neuroinflammation seen in both COVID-19 and epilepsy.

### 3.7. Drug Repositioning Prediction via LINCS L1000

We used the LINCS L1000 platform to predict compounds that could reverse the shared immune-related gene expression patterns. Based on the top-ranked hub genes, the analysis identified seven compounds with significant reversal scores (adjusted *p* < 0.05), many of which target core elements of cytokine signaling.

Among these, Gabapentin stood out as a promising candidate. It was predicted to affect *CX3CR1*, *TLR4*, and *SELPLG*—genes linked to microglial activation and immune cell trafficking—closely matching the immune pathways highlighted in our earlier analyses.

Other high-ranking compounds included Betamethasone, Retinol, Asiatic acid, and Bimatoprost, each targeting multiple inflammatory regulators (Table 1). These results offer a data-driven basis for exploring drug repurposing in neuroimmune conditions.

Gabapentin was selected for further evaluation due to its overlap with key immune pathways and existing preclinical evidence supporting its immunomodulatory effects (see Section 3.8 and Table 2).

### 3.8. Validation of Drug Efficacy and Mechanism

To evaluate Gabapentin’s potential as a repurposed therapy, we examined its predicted targets—*CX3CR1*, *TLR4*, and *SELPLG*—using ROC curve analysis and gene expression profiles.

**Figure 8:**
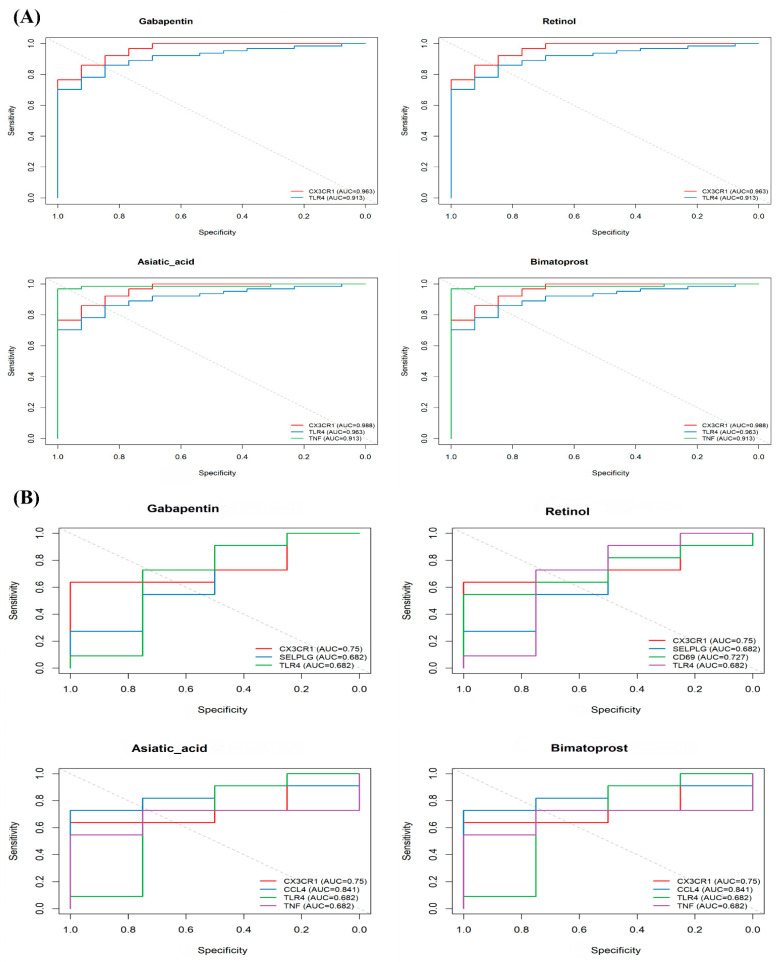
Transcriptional validation of candidate drug targets. (**A**) ROC curves for Gabapentin, Retinol, Asiatic acid, Betamethasone, and Bimatoprost targets in the epilepsy dataset (GSE256068). (**B**) ROC analysis in the COVID-19 dataset (GSE149689), showing reduced classification accuracy.

In the epilepsy dataset (GSE256068), these genes showed strong classification performance, with AUC values above 0.90 (Figure 8A). In contrast, their predictive power in the COVID-19 dataset (GSE149689) was lower. For example, CD69 showed an AUC of only 0.568 (Figure 8B), possibly reflecting greater immune heterogeneity in COVID-19 brains.

Importantly, *CX3CR1* and *TLR4* have also been validated in experimental studies. Gabapentin has been shown to reduce *CX3CL1*-induced microglial activation in rodent pain models [17], and to suppress TLR4–NF-κB signaling in models of chronic inflammation [18].

Together, these findings suggest that Gabapentin is a promising candidate for treating immune-related epilepsy, with support from both transcriptomic predictions and prior biological evidence.

### 3.9. Mechanistic Model of Gabapentin’s Dual Action in Neuroinflammation

**Figure 9:**
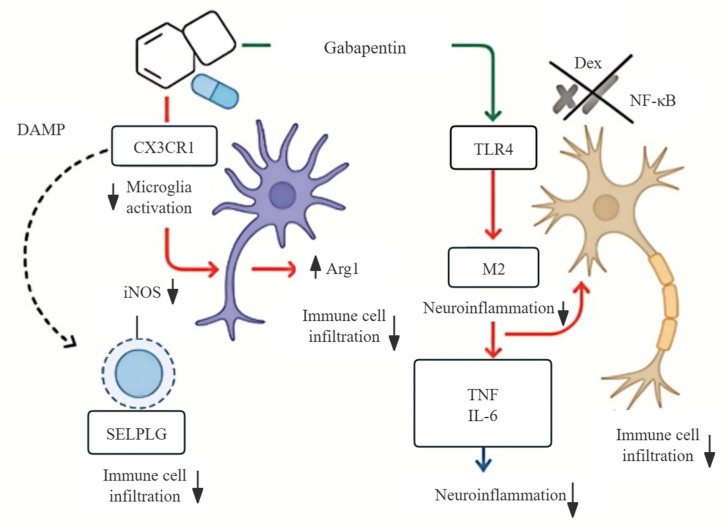
Schematic model illustrating the neuroimmune mechanism of Gabapentin. Gabapentin inhibits microglial CX3CR1 and neuronal TLR4 signaling, suppressing danger-associated molecular patterns (DAMP)-induced M1 polarization and promoting M2-like phenotypes. These effects result in reduced proinflammatory cytokine production and immune cell infiltration. Compared to dexamethasone, Gabapentin shows neural specificity with potentially fewer off-target effects.

To illustrate how Gabapentin might regulate neuroinflammation, we developed a schematic model based on transcriptomic data together with supporting literature (Figure 9). Our analysis suggests that Gabapentin could inhibit two key receptors involved in innate immune signaling, namely CX3CR1 on microglia and TLR4 on neurons. Inhibiting these pathways may influence microglial polarization. Traditionally, activated microglia have been described in terms of two phenotypes: M1-like cells, which typically express inducible nitric oxide synthase (iNOS) and release pro-inflammatory mediators, and M2-like cells, which express arginase-1 (Arg1) and are thought to contribute to anti-inflammatory and repair processes. In our dataset, Gabapentin appeared to reduce iNOS while increasing Arg1 expression, which is generally taken as a shift toward an anti-inflammatory profile.

It should be noted, however, that the classical M1/M2 framework has been increasingly recognized as an oversimplification. Recent work indicates that microglia display a broad spectrum of intermediate and context-dependent states, reflecting the complexity of neuroimmune responses. In addition to polarization, Gabapentin may also reduce immune cell infiltration through modulation of SELPLG, and may dampen the production of cytokines such as TNF and IL-6. Overall, these findings are consistent with a more targeted, CNS-directed immunomodulatory role of Gabapentin, in contrast to the broad systemic suppression observed with corticosteroids such as dexamethasone.

These transcriptomic predictions are corroborated by findings from the literature (see Table 2 for details). The data suggest that Gabapentin may offer a targeted, immune-modulating effect in disorders like epilepsy and post-COVID-19 neurological syndromes.

**Table 2:** Experimental and clinical studies supporting Gabapentin’s neuroimmune actions in epilepsy and post-COVID-19 conditions.

Study (Reference)	Model/System	Gabapentin Target/Pathway	Findings	Relevance
Yang et al. [17]	Arthritis model + microglia	CX3CL1–CX3CR1	Reduced CX3CL1 expression and microglial activation	Supports immune modulation
Rossi et al. [19]	Pilocarpine epilepsy model	α_2_δ voltage-gated calcium channel (VGCC), microglial response	Reduced microgliosis, increased seizure threshold	Confirms antiseizure role
Deng et al. [20]	Meta-analysis of 53 RCTs	—	RR = 2.30 for seizure reduction	High clinical relevance
Lee et al. [21]	Neuropathic pain model	IL-10 ↑, TNF/IL-6 ↓	Suppressed proinflammatory cytokines	Aligns with transcriptomic targets
Mcwilliam et al. [22]	Post-COVID-19 neuropathic pain	—	Pain relief and sensory recovery	Clinical support in post-COVID-19
Soltani et al. [23]	COVID-19 cough RCT	—	Reduced cough severity with Gabapentin + Montelukast	Functional neuromodulation
Tharakan et al. [24]	Long COVID cognitive symptoms	—	Mixed effects on sensory/cognitive outcomes	Risk–benefit insights
Garcia et al. [25]	RCT for COVID-19-related parosmia	—	67% benefit in early series; RCT neutral	Suggests limited benefit

Note: References are listed in detail in the main manuscript reference list.

## 4. Discussion

Epilepsy and COVID-19 represent two clinically and pathophysiologically distinct conditions—one a chronic neurological disorder characterized by recurrent seizures and neuronal hyperexcitability, the other an acute systemic viral syndrome marked by cytokine-driven inflammation [26,27]. Despite these differences, accumulating clinical evidence and transcriptomic studies have revealed surprising points of convergence: Both diseases are frequently associated with seizures, cognitive dysfunction, and widespread immune dysregulation involving both innate and adaptive pathways [13,28,29]. This study provides, for the first time, an integrative transcriptomic perspective on this overlap, identifying common immune-inflammatory signatures and prioritizing Gabapentin as a potential dual-action therapeutic through data-driven drug repositioning.

Our integrative transcriptomic analysis identified 25 DEGs that were consistently dysregulated across two independent platforms: scRNA-seq data from PBMCs of COVID-19 patients and bulk RNA-seq data from hippocampal tissue of epilepsy patients with TLE-HS. Among them, 22 genes were significantly upregulated and 3 were downregulated, representing a robust, cross-condition immune signature. Notably, these DEGs were enriched in canonical neuroimmune pathways, including cytokine–cytokine receptor interaction, NF-κB activation, and Toll-like receptor signaling. These pathways are well-established contributors to microglial priming, BBB disruption, and persistent leukocyte recruitment—all core features of both COVID-19 neuropathology and epilepsy-associated neuroinflammation [30,31,32].

Hub genes such as “*TNF*”, “*CX3CR1*”, “*CD69*”, and “*CCL3*” emerged as critical nodes driving immune cell adhesion, chemokine signaling, and glial activation [33,34,35,36]. In contrast, downregulated genes such as “*PECAM1*” and “*FCGR3A*”, known for their roles in endothelial integrity and immune clearance, point toward a suppressed resolution phase, possibly contributing to chronic neuroinflammatory persistence [37,38].

These findings collectively reflect a transcriptional landscape shaped by proinflammatory dominance coupled with impaired immune resolution—a hallmark of “non-resolving inflammation” increasingly recognized in chronic CNS conditions. Importantly, the recurrence of these gene expression changes across distinct data types and disease contexts supports their biological robustness and immunopathological relevance.

To further understand the immune imbalance reflected in the 25 shared DEGs, we conducted multi-angle functional interpretations of the hub genes, integrating prior literature from neuroinflammation, infectious disease, and immunoepileptology.

CX3CR1, the receptor for fractalkine (CX3CL1), is crucial for neuron–microglia signaling. In rodent epilepsy models, seizure activity induces upregulation of the FKN/CX3CR1 axis and microglial activation; pharmacological inhibition of CX3CR1 attenuates microglial activation and neurodegeneration [39]. Furthermore, fractalkine–CX3CR1 interaction has been implicated in seizure-induced microglial activation, further linking this pathway to neuroinflammatory seizure mechanisms [40]. In the context of COVID-19, peripheral immune profiling has revealed increased CX3CR1 expression on monocytes—suggesting enhanced chemotactic potential and neuroimmune crosstalk consistent with neuroinflammatory features observed in our transcriptomic analyses [41].

TLR4, a canonical pattern recognition receptor, is activated by both viral components and endogenous DAMPs such as HMGB1. In epilepsy, TLR4 signaling triggers glial activation and cytokine release via the MyD88–NF-κB pathway, enhancing neuronal excitability and BBB permeability [42,43]. In COVID-19, TLR4 upregulation correlates with systemic cytokine storm and lung–brain axis inflammation, positioning it as a shared upstream driver of immune amplification [44].

Conversely, PECAM1 (CD31) is a key mediator of endothelial integrity and immune effector trafficking. Its downregulation is linked to compromised vascular barrier function and impaired leukocyte transmigration resolution. Studies have shown that reduced PECAM1 expression exacerbates BBB leakage and immune cell infiltration in both viral encephalitis and seizure models [45]. Similarly, FCGR3A, downregulated here, plays a role in antibody-dependent cellular cytotoxicity and immune complex clearance—its deficiency may contribute to sustained inflammation and inefficient pathogen removal [46].

CD69, an early activation marker of T and NK cells, also functions as a regulator of lymphocyte retention and inflammatory resolution. Its upregulation in both datasets suggests persistent immune cell activation without appropriate resolution—a feature of chronic neuroinflammation [47].

Together, these findings reinforce the concept of immune polarization: a state where proinflammatory cytokine and chemokine pathways (e.g., *TNF*, *CXCL10*, *CCL3*) are upregulated, while genes associated with vascular repair and immune clearance (e.g., *PECAM1*, *FCGR3A*) are suppressed. This dual phenotype may represent a “non-resolving neuroimmune state,” a pathophysiological hallmark shared by chronic epilepsy and post-viral neuroinflammation.

Functional enrichment analyses further support this view. Upregulated DEGs were predominantly enriched in “cytokine–cytokine receptor interaction,” “NF-κB signaling,” and “Toll-like receptor signaling,” all of which orchestrate inflammatory amplification. Downregulated DEGs, in contrast, were associated with “NK cell–mediated cytotoxicity” and “leukocyte transendothelial migration,” suggesting defects in immune effector resolution. This imbalance illustrates a two-sided immune dysfunction, wherein activation dominates but resolution falters, promoting persistent inflammation and tissue vulnerability.

To explore upstream regulatory influences, we constructed a transcriptional regulatory network based on ENCODE ChIP-seq datasets. FOS and EGR2 emerged as proinflammatory transcriptional activators of CX3CR1, CD69, and CCL3. FOS, a component of the activator protein-1 (AP-1) complex, is rapidly induced during seizure activity and has been reported in epileptic brain tissue, supporting its role in cytokine induction [48]. RELA, in contrast, was linked to the regulation of downregulated immune clearance genes and is a known effector of NF-κB–mediated transcription in viral responses and glial suppression pathways [49]. The divergent activity of these TFs mirrors the transcriptional dichotomy observed in our DEG sets.

Among the compounds identified through LINCS L1000 perturbation reversal analysis, Gabapentin was predicted to target multiple hub genes within the shared immune-inflammatory network—including CX3CR1, TLR4, and SELPLG—suggesting a role that extends beyond its classical function as an antiepileptic modulator of the α2δ subunit of voltage-gated calcium channels (VGCCs).

Traditionally, Gabapentin’s antiseizure efficacy has been attributed to VGCC modulation, reducing presynaptic calcium influx and excitatory neurotransmitter release. Recent evidence, however, points to non-canonical immunomodulatory actions that may complement its antiepileptic properties. Targeting the TLR4–NF-κB axis reduces neuroinflammation and pain behaviors in rodent models [50], suggesting that agents with convergent downstream effects—potentially including Gabapentin—could modulate this pathway indirectly. It also downregulates CX3CR1, a receptor critical for microglial chemotaxis and activation in viral encephalitis and epilepsy [17]. By concurrently dampening these innate immune receptors, Gabapentin may mitigate both seizure-related neuroinflammation and COVID-19-induced immune activation.

In addition, Gabapentin has been reported to shift microglial phenotypes toward resolution. Specifically, it enhances IL-10 and Arg1 expression, markers of M2-like anti-inflammatory states, while reducing M1-like polarization [51]. This immunological reprogramming is consistent with our transcriptomic observation of transcriptional polarization and provides further support for Gabapentin’s dual role as both an antiseizure and an immunotherapeutic agent.

Clinically, Gabapentin is approved for partial-onset seizures, neuropathic pain, and fibromyalgia, with a favorable safety profile. Its indications overlap with symptoms commonly observed in post-COVID-19 neurological syndromes (e.g., headache, chronic pain, neuroinflammation), making it a plausible candidate for translational application in these contexts [51,52,53], but translational application requires caution. Emerging safety signals include Gabapentin-associated movement disorders in humans [54] and hippocampal vulnerability in rodent models under prolonged or high-dose exposure [55]. Interestingly, while preclinical models often emphasize beneficial effects, clinical reports in humans disproportionately highlight adverse neurological outcomes, underscoring the need for cautious translational extrapolation. These discrepancies underscore the need for dose-controlled validation and biomarker-guided clinical stratification. Although Gabapentin’s primary target is the α2δ subunit of VGCCs, Ca^2+^ influx through VGCCs can activate transcription factors such as NF-κB and AP-1. Thus, Gabapentin’s canonical mechanism may indirectly influence inflammatory cascades, although direct experimental validation remains limited.

Taken together, these findings suggest that Gabapentin’s repurposing potential lies in its dual capacity to reduce hyperexcitability and dampen maladaptive neuroinflammation. At the same time, carefully designed preclinical studies and biomarker-stratified clinical trials are required to balance therapeutic benefits against dose-related risks, particularly regarding hippocampal integrity and long-term safety.

### 4.1. Translational Potential and Clinical Implications

The repositioning of Gabapentin as a potential immunomodulatory agent opens promising translational avenues. Unlike traditional immunosuppressants such as corticosteroids, which often produce systemic side effects—including metabolic disturbances, increased infection risk, and neuropsychiatric complications—Gabapentin has a long-established safety record in treating CNS disorders, particularly partial-onset epilepsy and neuropathic pain. Its favorable pharmacokinetics, CNS selectivity, and oral formulation support its practical viability in clinical settings.

What makes Gabapentin particularly attractive is its ability to modulate microglial polarization and inflammatory signaling pathways (e.g., CX3CR1 and TLR4) without broadly suppressing systemic immunity. This selective action is especially valuable in chronic neuroinflammatory conditions, such as epilepsy and post-viral CNS syndromes, where immunological imbalance must be corrected without compromising neural surveillance.

Given its regulatory approval and clinical accessibility, Gabapentin could feasibly be integrated into future immunotherapy trials—particularly those guided by biomarkers like CSF cytokine profiles or translocator protein positron emission tomography (TSPO-PET) imaging patterns. Such trials would help refine patient selection, identify optimal dosing regimens, and clarify its place in the neuroimmune therapeutic landscape.

That said, several open questions remain. The precise immunological targets of Gabapentin are incompletely defined, and potential off-target effects may vary across disease states, cell types, and dosages. Moreover, although peripheral tolerability is well documented, its central immune effects may prove dose-sensitive, particularly in acute settings such as viral encephalitis. Importantly, clinical data on Gabapentin’s use during active CNS infection or seizure exacerbation remain sparse, underscoring the need for focused prospective evaluation.

### 4.2. Limitations and Future Directions

Our study provides a systems-level view of immune convergence in COVID-19 and epilepsy, yet several limitations merit attention.

First, our transcriptomic analysis relied on PBMC samples from COVID-19 patients and surgically resected hippocampal tissue from epilepsy patients, which may capture disease endpoints rather than dynamic changes. Second, although key gene networks were identified, we did not perform protein-level validation or functional assays to test their biological relevance. Third, drug repositioning was entirely computational in nature, and its predictive value requires experimental corroboration. Moreover, while our data suggest potential immunomodulatory effects of Gabapentin via CX3CR1 and TLR4, these roles remain hypothetical. Most evidence comes from transcriptomic predictions and limited preclinical observations, and the effective doses reported in rodent models of inflammation (typically 30–100 mg/kg) correspond to relatively high human equivalents compared with routine clinical practice.

To address these limitations, future studies should consider:

(1) Applying longitudinal transcriptomic profiling in early-stage COVID-19 and epilepsy models to capture time-resolved immune activity;
(2) Conducting mechanistic studies using CRISPR/Cas9-mediated knockout of *CX3CR1* and *TLR4* in microglia to assess Gabapentin’s specificity;
(3) Testing Gabapentin in preclinical models of virus-triggered or immune-mediated seizures;
(4) Designing stratified clinical trials in patients with neuroinflammatory signatures (e.g., elevated CX3CL1 or TSPO-PET hypermetabolism).

Combination therapies with Gabapentin and agents such as corticosteroids or TLR4 antagonists also warrant exploration, with careful attention to comorbidities like BBB disruption or cardiovascular disease, which may affect drug safety and response. Importantly, although preclinical data suggest anti-inflammatory effects, whether standard clinical doses used in epilepsy or neuropathic pain (approximately 900–3600 mg/day in humans) are sufficient to achieve CNS-specific immune modulation remains uncertain and requires dose-controlled validation in future studies.

## 5. Conclusions

This study identifies a convergent neuroimmune pathway—centered on the CX3CR1–TLR4–NF-κB axis—that is active in both COVID-19 and epilepsy, highlighting a shared inflammatory endophenotype. Our data reveal persistent cytokine activation paired with impaired resolution, suggesting a mechanistic basis for chronic neuroinflammation across these conditions.

Gabapentin emerged from our analysis as a plausible therapeutic candidate with both neuromodulatory and immunological potential. Its ability to modulate shared immune networks, as indicated by transcriptomic and pathway analyses, underscores its translational relevance.

Moving forward, rigorously designed clinical studies—preferably randomized, biomarker-stratified, and mechanistically informed—are essential to evaluate the safety, efficacy, and scope of Gabapentin in immune-mediated epilepsy and post-infectious CNS disorders. These efforts may ultimately pave the way toward precision immunotherapy in neuroinflammatory disease.

## Figures and Tables

**Table 1:** Predicted compounds capable of reversing the shared immune gene signature in COVID-19 and epilepsy, identified through LINCS L1000 perturbation analysis.

Compound	Adjusted *p*-Value	Combined Score	Targeted Genes
Bimatoprost	0.0024	670.68	*CX3CR1, CCL4, TLR4, TNF*
Retinol	0.0024	670.68	*CX3CR1, SELPLG, CD69, TLR4*
Betamethasone	0.0024	667.01	*CX3CR1, CCL4, CD69, TLR4*
Gabapentin	0.0082	295.44	*CX3CR1, SELPLG, TLR4*
Asiatic acid	0.0024	663.38	*CX3CR1, CCL4, TLR4, TNF*
N-benzylnaltrindole	0.037	139.42	*MPZL2*
Nitazoxanide	0.037	138.66	*MPZL2*

Note: Adjusted *p*-values and combined scores were derived from LINCS L1000 perturbation analysis. Targeted genes represent those involved in shared DEGs.

## Data Availability

Both datasets are publicly available from the Gene Expression Omnibus (GEO) database. GSE149689 [PMID: 35365380] (https://www.ncbi.nlm.nih.gov/geo/query/acc.cgi?acc=GSE149689, accessed on 28 March 2025) contains scRNA-seq data from peripheral blood mononuclear cells (PBMCs) of COVID-19 patients and healthy controls. GSE256068 [PMID: 38118286] (https://www.ncbi.nlm.nih.gov/geo/query/acc.cgi?acc=GSE256068, accessed on 28 March 2025) includes bulk RNA-seq data from hippocampal tissues of patients with TLE-HS and healthy controls.

## Supplementary Materials

The following supporting information can be downloaded at: https://www.mdpi.com/article/10.3390/biomedicines13092133/s1, Supplementary File S1: Common upregulated DEGs with statistical details; Supplementary File S2: Common downregulated DEGs with statistical details; Supplementary File S3: Complete GO enrichment results; Supplementary File S4: Complete KEGG enrichment results; Supplementary File S5: STRING edge table for upregulated PPI network; Supplementary File S6: STRING edge table for downregulated PPI network; Supplementary File S7: cytoHubba (MCC) hub-gene rankings; Supplementary File S8: TF–gene regulatory network table for upregulated hub genes; Supplementary File S9: TF–miRNA regulatory network table for downregulated hub genes.

## Abbreviations

AP-1	activator protein-1
BBB	blood–brain barrier
DAMP	damage-associated molecular pattern
DEG	differentially expressed gene
DSigDB	Drug Signatures Database
GEO	Gene Expression Omnibus
HMGB1	high-mobility group box 1
ITGAX	integrin subunit alpha X (CD11c)
MCC	Maximal Clique Centrality
SELPLG	selectin P ligand (PSGL-1)
TLE-HS	temporal-lobe epilepsy with hippocampal sclerosis
TSPO-PET	translocator protein positron emission tomography
VGCC	voltage-gated calcium channel
